# Associations of Health Care System Deficiency–Related Patient Deterioration With Burnout, Depressive Symptoms, and Sleep Problems Among Emergency Medical Service Providers: Cross-Sectional Study

**DOI:** 10.2196/90130

**Published:** 2026-07-27

**Authors:** Min Seung Choi, Soo Jin Kim, Yonghwan Moon, Hyekyung Woo

**Affiliations:** 1 Department of Emergency Medical Services, Graduate School of Public Health & Welfare, Eulji University, Seongnam, Republic of Korea; 2 Laboratory of Emergency Medical Services, Seoul National University Hospital Biomedical Research Institute, Seoul, Republic of Korea; 3 Department of Health Administration, Kongju National University, 56 Gongjudaehak-ro, Kongju-si, 32588, Republic of Korea, 82-41-850-3486; 4 Division of Research Planning, National Center for Mental Health, Mental Health Research Institute, Seoul, Republic of Korea

**Keywords:** patient deterioration, health care service disruptions, burnout, depression, sleep disorders, emergency medical technicians

## Abstract

**Background:**

On February 6, 2024, the Korean government announced an increase in medical school enrollment quotas, triggering a nationwide strike by medical residents that precipitated a severe health care crisis. Prehospital emergency medical service (EMS) providers were forced to transport patients to distant hospitals, resulting in some patients experiencing symptom deterioration or death due to delayed critical care. Recent studies have examined moral injury—a mental health response to events that undermine moral values—among EMS providers, with exposure to potentially morally injurious events increasing the likelihood of depression, anxiety, and posttraumatic stress disorder. However, studies specifically examining the association between health care–related patient deterioration experiences caused by health care service disruptions and mental health outcomes among EMS providers remain limited.

**Objective:**

This study evaluated the associations of patient deterioration due to health care service disruptions (hereafter referred to as health care–related patient deterioration experiences [HPDEs]) with burnout, depressive symptoms, and sleep problems among prehospital EMS providers during government–medical community disputes.

**Methods:**

We conducted a retrospective cross-sectional survey from November 1 to December 31, 2024, among EMS providers across South Korea using a web-based questionnaire. The independent variable was the presence or absence of HPDE, and the dependent variables were 3 mental health outcomes (personal burnout [PB], work-related burnout [WRB], and citizen-related burnout [CRB]), depressive symptoms, and sleep problems. *χ*^2^ tests and multivariable logistic regression were used to assess associations and identify influencing factors after adjustment for potential confounders.

**Results:**

Of 845 participants, 75% (n=626) reported HPDEs, which were associated with higher odds of PB, WRB, CRB, depressive symptoms, and sleep problems. Additionally, sex, age, educational attainment, and work region significantly influenced burnout, depressive symptoms, and sleep problems.

**Conclusions:**

During government–medical community disputes, EMS providers exposed to HPDEs—a form of potentially morally injurious event resulting from emergency medical resource shortages—had significantly elevated odds of burnout, depressive symptoms, and sleep problems. These findings underscore the urgent need for systematic psychological interventions and regular mental health screenings, such as for moral injury, to mitigate adverse mental health outcomes during future health care service disruptions.

## Introduction

On February 6, 2024, the Korean government announced an increase in medical school enrollment quotas to secure essential health care personnel. In response, beginning February 20, approximately 12,000 medical residents—more than 90% of all residents—initiated a strike by submitting their resignations [1]. This led to a sharp reduction in essential medical personnel across hospitals nationwide, including emergency medical centers, disrupting both the broader health care system and the emergency medical system severely.

Prehospital emergency medical service (EMS) providers were particularly affected. Although these providers are required to transport emergency patients to the nearest available hospital after administering initial treatment [2], many were frequently forced to seek hospitals farther away when nearby facilities could not accept patients. Emergency medical centers nationwide report bed availability and staffing levels in real time to the National Emergency Medical Center through the National Emergency Department Information System, and EMS providers use this data to select transport destinations. When a facility is unable to accept emergency patients due to staff shortages or other reasons, a “care restriction message” is displayed on the dashboard. In 2024, a total of 110,033 care restriction messages were recorded on the National Emergency Medical Center’s integrated dashboard—an 88% increase compared to 58,520 cases in 2023, prior to the crisis. Notably, care restrictions attributed to staff shortages surged to 43,658 cases, representing a 132.8% increase over the previous year [3]. During this process, some patients experienced symptom deterioration or death due to delays in receiving critical emergency care that should have been promptly administered at hospitals. EMS providers witnessed these outcomes firsthand [4,5].

Recently, moral injury (MI) has been studied as a mental health issue among EMS providers and other health care professionals [6-8]. MI is a response to events that undermine an individual’s beliefs and moral values [6,9], triggered by exposure to repetitive or severe potentially morally injurious events (PMIEs) [10]. Individuals exposed to PMIEs are 2.6 times more likely to develop mental health disorders such as depression, anxiety, and posttraumatic stress disorder (PTSD) compared with those who are not exposed [11]. EMS providers must make instantaneous decisions in life-and-death situations and often find themselves taking actions that conflict with their moral beliefs [12]. Due to the nature of their work, they are repeatedly exposed to circumstances that can trigger MI [8]. Frequent exposure to PMIEs increases the risk of developing MI [10,13,14]. Its core features—guilt, shame, and feelings of betrayal toward institutions or leadership—not only contribute to mental health issues such as depression and anxiety [15] but also carry significant occupational and social consequences. EMS providers who experience MI report stronger intentions to leave the profession [16], along with increased rates of medical incidents and declines in care quality [17]. Understanding EMS providers’ exposure to PMIEs and their association with mental health outcomes is therefore essential for prevention and intervention.

During health care service disruptions, such as the COVID-19 pandemic and government–medical community disputes, emergency medical resources may become scarce and hospitals may be unable to accommodate incoming patients. In these contexts, EMS providers often face prolonged prehospital treatment times and witness patient deterioration helplessly. This study defines such specific and clinically critical incidents—in which EMS providers fail to transport patients in a timely manner due to health care service disruptions and are forced to witness patient deterioration—as health care–related patient deterioration experiences (HPDEs). While PMIEs represent a broad psychological concept encompassing all events that violate an individual’s moral beliefs, HPDEs constitute a specific, occupationally distinct type of event that EMS providers may experience as PMIEs [18].

Although recent research has begun to explore MI among EMS providers and health care professionals, studies specifically examining the association between HPDE exposure and mental health outcomes remain limited. Accordingly, we evaluated associations of HPDEs with personal burnout (PB; the degree of physical and psychological fatigue and exhaustion experienced by an individual), work-related burnout (WRB; the degree of physical and psychological fatigue and exhaustion an individual feels in relation to their work), citizen-related burnout (CRB; the degree of physical and psychological fatigue and exhaustion an individual feels in relation to work involving interactions with citizens, ie, clients), depressive symptoms, and sleep problems among EMS providers in South Korea during health care service disruptions.

## Methods

### Study Design and Population

This was a retrospective cross-sectional study. The survey was designed as an open web-based survey accessible to any EMS provider through a distributed link. Among the 14,310 EMS providers working in South Korea, 855 voluntarily participated in the survey, representing 6% of the total population. After excluding 1 participant who did not consent to participate and 9 participants with missing data regarding age and years of service, 845 participants were included in the analysis.

### Data Source and Collection

The survey was conducted over a 2-month period from November 1 to December 31, 2024, targeting EMS providers nationwide through an online questionnaire. To account for internet security access issues at some provincial fire departments, both Naver and Google platforms were used in parallel. To reflect the working environment and health status of EMS providers during health care service disruptions caused by government–medical community disputes, as well as practical issues in emergency medical care, the validity of the questionnaire was reviewed through literature searches and interviews with field experts in emergency medical services, including EMS providers, emergency dispatching and operating centers, and a prehospital EMS policy officer. The final survey items were refined after the content had been modified through a pilot study.

The questionnaire, titled Korean EMS Provider’s Working Environment in Situations of Medical Disruption, included items regarding the following five domains:

1. Experience with prehospital emergency medical activities related to health care service disruptions (transport difficulties, delayed transfer times, and exposure to HPDEs)
2. Work life (workplace social support, organizational culture, and job satisfaction)
3. Mental health (psychological well-being, psychological stress, work-related exhaustion, burnout, depressive symptoms, suicidal ideation, and sleep problems)
4. General health status (self-assessed health and dietary habits)
5. Demographic and sociological characteristics of the study population (sex, age, marital status, and work region)

To increase response rates, participation was encouraged through official letters, standardized study information sheets, and participation links sent to institutions employing EMS providers and firefighter unions. Institutional coordinators distributed these links to their affiliated EMS providers via emails and SMS messages. EMS providers who received the link reviewed the study information and voluntarily decided whether to participate. To prevent duplicate responses, participation was restricted to once per person. Furthermore, the survey was designed so that data were saved to the server only after participants responded to all items and clicked the Submit button, meaning incomplete responses were automatically excluded from the analysis.

### Variables and Measures

The independent variable was the presence or absence of HPDEs, whereas the 3 dependent variables were burnout, depressive symptoms, and sleep problems. The measurement methods for each variable were as follows.

#### HPDE

In this study, an HPDE was operationally defined as the specific clinical experience of EMS providers who are forced to helplessly witness patient deterioration due to structural deficiencies in the health care system and delays in care. HPDEs refer to EMS providers who witness patient deterioration due to health care–related delays in care, corresponding to “omission” in the PMIE classification [18]. In the survey, participants were asked whether they had ever witnessed patient deterioration due to a health care system failure resulting from health care service disruption caused by government–medical community disputes that occurred after March 2024. Responses were categorized as “yes” for having such an experience at least once and “no” for not having that experience.

#### Burnout

Burnout was assessed using the Korean version of the Copenhagen Burnout Inventory [19], which consists of 3 domains: PB, WRB, and CRB [20]. For each item, participants could choose from 5 options: “always/almost always” (100 points), “often/very much” (75 points), “sometimes/somewhat” (50 points), “seldom/a little bit” (25 points), and “never/very little” (0 points). Average scores were calculated for each domain; higher scores indicated higher levels of burnout. An average score of 50 points or higher in each domain was considered indicative of burnout [21]. The Cronbach α for burnout was 0.96.

#### Depressive Symptoms

Depressive symptoms were assessed using the Center for Epidemiologic Studies Depression Scale, which measures experiences over the past week and consists of 20 items. For each item, respondents could choose from “less than 1 day per week,” “1‐2 days per week,” “3‐4 days per week,” or “5 or more days per week.’” Responses were converted into scores of 0 to 3 points according to the items. When the total score for the 20 items was 16 points or higher, the respondent was considered to have depressive symptoms [22]. The Cronbach α for depressive symptoms was 0.93.

#### Sleep Problems

Sleep problems were assessed by evaluating how frequently sleep-related issues occurred over the prior 12 months. The survey included 3 questions, with response options of “daily,” “several times a week,” “several times a month,” “rarely,” and “never.” Each response was converted into 0 to 4 points, and respondents with a total score of 6 points or higher were considered to have experienced sleep problems [23]. The Cronbach α for sleep problems was 0.86.

### Statistical Analysis

Continuous variables were reclassified into categorical boundaries for analysis; specifically, age was grouped into 5- to 10-year intervals (20‐29, 30‐34, 35‐39, 40‐44, and ≥45 years), and years of service were grouped into specific intervals (<4, 5‐9, 10‐14, and ≥15 years). All categorical variables are presented as frequencies and percentages (n, %). *χ*^2^ tests were performed to examine differences between participant characteristics and dependent variables. Logistic regression was conducted to determine odds ratios and 95% CIs for burnout, depressive symptoms, and sleep problems according to HPDE exposure status. In building the multivariable model, key demographic and occupational variables frequently used in previous studies of EMS providers and health care workers were comprehensively included as potential confounders. To prevent multicollinearity issues beforehand, correlations among covariates were examined. As a result, the correlation coefficients among age, years of service, and position were 0.7 or higher; therefore, only age—the variable that best reflects the representativeness of the model—was included. Additionally, after constructing the final multivariable model, multicollinearity was diagnosed by calculating the variance inflation factor (VIF), and it was confirmed that there was no multicollinearity issue as all VIF values were less than 5. Furthermore, since this study aimed to estimate the independent main effects of HPDE exposure, potential interactions between the primary independent variable and covariates were exploratorily evaluated. Although significant interactions were observed in some models, a main effects model excluding interaction terms was adopted as the primary analysis in the final multivariable model to focus on the primary hypothesis of main effects and to maintain the intuitiveness and consistency of result interpretation across the 5 dependent variable models. However, the identified interaction results are described as exploratory findings in the Results section. The final regression model included sex, age, marital status, household size, educational level, household income, job position, current assignment, work type, and work region as confounders. The goodness-of-fit of the multivariable logistic regression model was evaluated using the Hosmer-Lemeshow test and *χ*^2^ tests; *c* statistics were measured to assess the discriminative ability of the final model. Analyses were performed using SAS (version 9.4; SAS Institute), and the statistical significance level was set at *P*<.05.

### Ethical Considerations

This study was approved by the institutional review board of Eulji University (EUIRB2024–102). Before beginning the survey, all participants were provided with detailed information regarding the study purpose, procedures, data collection, and academic use. Electronic written informed consent was obtained from those who voluntarily agreed to participate and consented to the academic use of their data. The collected data were processed to be untraceable and unidentifiable to ensure the anonymity and confidentiality of the participants. All participants were recorded solely by a unique identification number assigned in the order of their participation. Research data were stored in encrypted files on a secure server with restricted access, and strict security measures were implemented to prevent anyone other than the research team from accessing the data. As a token of appreciation for survey participation, a small mobile beverage coupon was provided. The provision of contact information was optional and collected only from participants who wished to receive compensation. Ten participants were randomly selected from those who voluntarily provided their contact information to receive coupons. The collected contact information was managed separately from the anonymous research data and safely discarded once the purpose of compensation was fulfilled.

## Results

**Figure 1.**
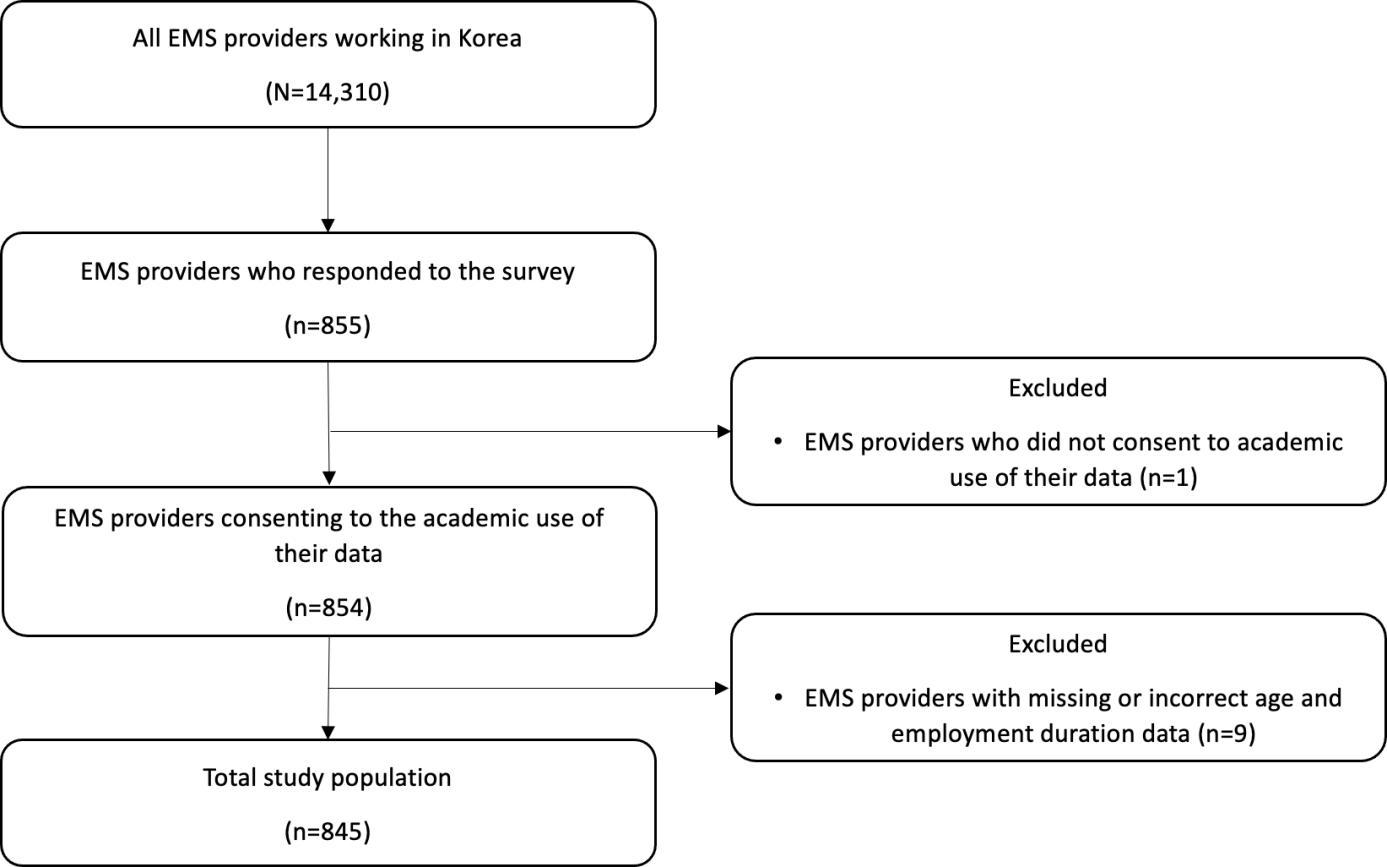
Flowchart of the study population selection among emergency medical service (EMS) providers in South Korea (November-December 2024).

Among the 845 study participants (Figure 1), 75% (n=626) reported HPDEs, which differed significantly by household size, current duties, and work region. The prevalences of mental health–related symptoms among all participants were as follows: PB 50.1% (n=423), WRB 46.9% (n=396), CRB 52.8% (n=446), depressive symptoms 19.8% (n=167), and sleep problems 46.8% (n=395). All of these outcomes were more prevalent in the HPDE group, with statistically significant differences (Table 1).

**Table 1.** Demographic characteristics, occupational factors, and mental health status of emergency medical service (EMS) providers by health care–related patient deterioration experience (HPDE) in South Korea (November-December 2024).

Potential risk factors	Total (N=845), n (%)	HPDE (n=626), n (%)	No HPDE (n=219), n (%)	*P* value
Sex				.93
Male	635 (75.2)	470 (75.1)	165 (75.5)	
Female	210 (24.8)	156 (24.9)	54 (24.5)	
Age				.12
20‐29	109 (12.9)	86 (13.7)	23 (10.5)	
30‐34	335 (39.6)	234 (37.4)	101 (45.9)	
34‐39	184 (21.8)	144 (23.0)	40 (18.2)	
40‐44	154 (18.2)	112 (17.9)	42 (19.1)	
≥45	63 (7.5)	50 (8.0)	13 (6.3)	
Marital status				.52
Single	311 (36.8)	239 (38.2)	72 (32.9)	
Married	511 (60.5)	371 (59.2)	140 (63.9)	
Divorced, separated, or widowed	15 (1.8)	10 (1.6)	5 (2.3)	
Declined to answer	8 (0.9)	6 (1.0)	2 (0.9)	
Household size				.02
1 person	177 (20.9)	145 (23.2)	32 (14.6)	
2 persons	206 (24.3)	142 (22.7)	64 (29.2)	
3 persons	234 (27.9)	164 (26.1)	70 (32.0)	
4 persons	188 (22.2)	145 (23.2)	43 (19.6)	
5 or more persons	40 (4.7)	30 (4.8)	10 (4.6)	
Education level				.08
High school or less	24 (2.8)	21 (3.4)	3 (1.4)	
College	800 (94.8)	586 (93.6)	214 (98.2)	
Graduate school	18 (2.1)	17 (2.7)	1 (0.2)	
Declined to answer	3 (0.3)	2 (0.3)	1 (0.2)	
Household income (million ₩^d^)				.72
<40	90 (10.7)	72 (11.5)	18 (8.2)	
40‐49.99	156 (18.5)	119 (19.0)	37 (16.9)	
50‐59.99	181 (21.4)	133 (21.2)	48 (21.9)	
60‐69.99	110 (13.0)	79 (12.6)	31 (14.2)	
≥70	255 (30.2)	185 (29.6)	70 (32.0)	
Declined to answer	53 (6.2)	38 (6.1)	15 (6.8)	
Years of service				.23
<4	304 (35.9)	218 (34.8)	86 (39.1)	
5‐9	292 (34.5)	214 (34.2)	78 (35.6)	
10‐14	140 (16.6)	105 (16.8)	35 (15.9)	
≥15	109 (13.0)	89 (14.2)	20 (9.4)	
Job rank				.47
*Sobang-sa*^a^ (firefighter)	255 (30.1)	181 (28.9)	74 (33.6)	
*Sobang-gyo* (fire engineer)	254 (30.0)	191 (30.5)	63 (28.6)	
*Sobang-jang* (fire lieutenant)	255 (30.1)	190 (30.4)	65 (29.5)	
*Over sobang-wi* (fire captain)	81 (9.8)	64 (10.2)	17 (8.3)	
Job position				.99
Ambulance crew	651 (77.0)	482 (77.0)	169 (76.8)	
Ambulance driver	164 (19.5)	122 (19.5)	42 (19.5)	
Other^b^	30 (3.5)	22 (3.5)	8 (3.7)	
Current assignment				.03
General EMS unit	619 (73.3)	445 (71.1)	174 (79.5)	
Special EMS unit	213 (25.2)	169 (27.0)	44 (20.0)	
EMS command and control center	13 (1.5)	12 (1.9)	1 (0.5)	
Work type				.31
Day shift	15 (1.8)	9 (1.5)	6 (2.7)	
Shift work (3 teams, 2 shifts)	450 (53.2)	340 (54.3)	110 (50.2)	
Other shift work^c^	380 (45.0)	277 (44.2)	103 (47.1)	
Work region				.003
Urban	137 (16.2)	113 (18.1)	24 (11.0)	
Suburban	343 (40.6)	262 (41.9)	81 (37.0)	
Rural	365 (43.2)	251 (40.0)	114 (52.0)	
Personal burnout				<.001
Yes	423 (50.1)	345 (55.1)	78 (35.9)	
No	422 (49.9)	281 (44.9)	141 (64.1)	
Work-related burnout				<.001
Yes	396 (46.9)	331 (52.9)	65 (30.0)	
No	449 (53.1)	295 (47.1)	154 (70.0)	
Citizen-related burnout				<.001
Yes	446 (52.8)	368 (58.8)	78 (35.9)	
No	399 (47.2)	258 (41.2)	141 (64.1)	
Depressive symptoms				<.001
Yes	167 (19.8)	142 (22.7)	25 (11.4)	
No	678 (80.2)	484 (77.3)	194 (88.6)	
Sleep problems				.001
Yes	395 (46.8)	313 (50.0)	82 (37.7)	
No	450 (53.2)	313 (50.0)	137 (62.3)	

a Lowest.

b Other includes tasks such as report reception, counseling, and administrative duties.

c Other shift work includes 4 teams and 2 shifts.

d 1 ₩=US $0.00067 as of July 15, 2026

Health care service disruptions were categorized into 3 domains: EMS providers’ workload burden, fire service support, and experiences of verbal violence (Table 2). In the workload burden domain, 98.8% (n=835) encountered difficulty arranging hospital transport, whereas 95.5% (n=807) faced refusal from nearby hospitals and were consequently required to transfer patients to facilities located more than 10 km away. Among these transfers, 89.6% (n=758) occurred at a rate of 1 to 2 long-distance transports per day on average. Notably, EMS providers performing 5 or more long-distance transports daily exhibited more than a 10-fold increase in the odds of HPDE exposure compared with those performing fewer transports. Furthermore, 17.7% (n=150) experienced waiting times for hospital selection that exceeded predisruption conditions by more than 1 hour, and 95.3% (n=805) reported communication difficulties with hospital medical staff. Together, these findings indicate that increasing long-distance transfer frequencies and the resulting workload burden are significantly associated with elevated HPDE exposure likelihood.

In a multivariable logistic regression adjusted for sociodemographic variables, HPDE exposure was associated with significantly higher odds of mental health outcomes (Table 3). Among sociodemographic characteristics, sex and work region were significantly correlated with burnout, depressive symptoms, and sleep problems. Female providers showed elevated odds of PB, WRB, and depressive symptoms. Compared with rural areas, suburban providers demonstrated higher odds of PB, WRB, CRB, and sleep problems, whereas urban providers exhibited the greatest odds. Additionally, participants aged 30 to 34 years had higher odds of depressive symptoms. Detailed analysis results for all confounders included in the model are presented in Multimedia Appendix 1. Furthermore, exploratory analysis of interactions between the primary independent variable and covariates revealed no significant interactions in the 3 burnout models; however, significant interactions with HPDE exposure were observed for sex in the depressive symptom model (*P*=.007) and for age group in the sleep problem model (*P*=.046). Nevertheless, these interaction effects represented minor findings limited to only a few models. To clearly convey the independent main effects of HPDE exposure on the entire paramedic population—which is the primary objective of this study—and to maintain consistency in interpreting all models, the final results were presented based on the main effects model.

**Table 2.** Association between health care service disruption characteristics and health care–related patient deterioration experiences (HPDEs) among emergency medical service (EMS) providers in South Korea (November-December 2024).

Variable	Total (N=845), n (%)	HPDE (n=626), n (%)	No HPDE (n=219), n (%)	*P* value
Workload				
Difficulty in hospital selection				<.001
Yes	835 (98.8)	624 (99.7)	211 (96.4)	
No	10 (1.2)	2 (0.3)	8 (3.6)	
Long-distance transfer due to nearby hospital refusal				<.001
Yes	807 (95.5)	613 (97.9)	194 (75.0)	
No	38 (4.5)	13 (2.1)	25 (25.0)	
Average daily long-distance transfers				<.001
1‐2 times	506 (59.8)	362 (57.8)	144 (65.8)	
3‐4 times	161 (19.0)	140 (22.4)	21 (9.6)	
≥5 times	91 (10.8)	83 (13.3)	8 (3.7)	
Not applicable	87 (10.4)	41 (6.5)	46 (20.9)	
Delay over 1 hour for hospital selection				<.001
No	72 (8.5)	30 (4.8)	42 (19.1)	
<30%	365 (43.3)	244 (39.0)	121 (55.5)	
30%‐50%	258 (30.5)	217 (34.7)	41 (18.6)	
≥50%	150 (17.7)	135 (21.5)	15 (6.8)	
Prolonged return-to-base time				<.001
<1 hour	465 (55.0)	304 (48.6)	161 (73.5)	
≥1 hour	380 (45.0)	322 (51.4)	58 (26.5)	
Private ambulance request at scene				<.001
Yes	249 (29.4)	216 (34.5)	33 (15.0)	
No	596 (70.6)	410 (65.5)	186 (85.0)	
Reason for increased direct medical control				<.001
No increase	200 (23.6)	103 (16.5)	97 (44.3)	
Increased for critical patient emergency care	144 (17.0)	114 (18.2)	30 (13.7)	
Increased for hospital selection	312 (36.9)	250 (39.9)	62 (28.3)	
Increased for other reasons	189 (22.5)	159 (25.4)	30 (13.7)	
Urgent administrative task experience				<.001
Yes	661 (78.2)	534 (85.3)	127 (58.0)	
No	184 (21.8)	92 (14.7)	92 (42.0)	
Communication difficulty with hospital staff				<.001
Yes	805 (95.3)	620 (99.0)	185 (84.5)	
No	40 (4.7)	6 (1.0)	34 (15.5)	
Fire department institutional support				
Sufficiency of fire department institutional support				<.001
Insufficient	639 (75.6)	515 (82.3)	124 (56.6)	
Neutral	178 (21.1)	97 (15.5)	81 (37.0)	
Sufficient	28 (3.3)	14 (2.2)	14 (6.4)	
Most needed stage for fire department support				.97
From call to on-scene arrival	109 (12.9)	80 (12.8)	29 (13.2)	
From patient contact to hospital selection	691 (81.8)	513 (81.9)	178 (81.3)	
In other areas	45 (5.3)	33 (5.3)	12 (5.5)	
Verbal abuse from patients or caregivers				<.001
Yes	490 (58.0)	416 (66.5)	74 (34.1)	
No	355 (42.0)	210 (33.5)	145 (65.9)	

**Table 3.** Multivariable logistic regression analysis of factors associated with mental health problems among emergency medical service (EMS) providers in South Korea (November-December 2024). Adjusted for covariates including sex, age, marital status, household size, education level, household income, job position, current assignment, work type, and work region. The full regression model including all covariates is available in Table S1 in Multimedia Appendix 1. AOR: adjusted odds ratio; HPDE: health care–related patient deterioration experience.

HPDE^b^	Personal burnout, AOR^a^ (95% CI)	Work-related burnout, AOR (95% CI)	Citizen-related burnout, AOR (95% CI)	Depressive symptoms, AOR (95% CI)	Sleep problems, AOR (95% CI)
No	Reference	Reference	Reference	Reference	Reference
Yes	2.05 (1.46-2.87)	2.66 (1.86-3.78)	2.74 (1.95-3.86)	2.22 (1.36-3.60)	1.63 (1.17-2.27)

a AOR: adjusted odds ratio.

b HPDE: health care system–related patient deterioration experience.

## Discussion

This study showed that HPDEs among EMS providers during health care service disruptions have significant associations with burnout, depressive symptoms, and sleep problems. To understand the mechanism through which these clinical experiences inflict severe psychological impacts on EMS providers, it is necessary to interpret HPDEs within the theoretical framework of PMIEs.

PMIEs were first classified by Litz et al [10], who identified 3 common categories: commission, omission, and betrayal. Commission refers to actions that directly violate one’s moral beliefs or values; omission denotes the failure to perform actions one believes to be morally right, often due to situational constraints; and betrayal occurs when one feels let down by others, such as supervisors or colleagues, who act against one’s moral convictions or compel unethical behavior [18]. In this context, HPDEs—the core variable of this study—can be interpreted as complex PMIEs that encompass both the emotion of omission, which stems from failing to fulfill the duty of patient protection due to situational constraints, and the emotion of betrayal, rooted in the feeling that the state and health care system have neglected both EMS providers and patients [8,24,25]. During the strike, most EMS providers surveyed were unable to secure nearby receiving hospitals and were forced to transport patients to distant facilities, resulting in approximately 75% witnessing patient deterioration helplessly. These repeated experiences of long-distance transport and patient deterioration do not stem from a lack of individual competence among EMS providers, but rather originate from uncontrollable structural limitations. The repeated experience of having the primary mission of saving patient lives through rapid and effective treatment blocked by external factors ultimately undermines their moral beliefs and professional values.

EMS providers working in urban areas exhibited a higher prevalence of mental health issues, including burnout, depressive symptoms, and sleep problems, likely due to shift work and increased call volumes. Shift work, a common scheduling pattern among EMS personnel, contributes to both physical and psychological strain; populations engaged in shift work demonstrate elevated rates of depressive symptoms, sleep problems, anxiety, and fatigue compared with the general population [26,27]. Urban regions experience frequent late-night emergency incidents due to high population density and diverse lifestyle patterns, disproportionately exposing EMS providers to violent encounters and psychiatric emergencies. Frequent nighttime deployments impair sleep quality, whereas repeated exposure to high-risk scenes sustains heightened psychological arousal, thereby threatening overall mental health. The findings of this study support the conclusions of previous research that these unique urban occupational characteristics and heavy workloads act as core risk factors for PTSD, depressive symptoms, anxiety, burnout, and sleep problems [28].

Due to the nature of their work, EMS providers exhibit higher prevalence rates of mental health disorders compared with the general population, even in the absence of special situations such as the COVID-19 pandemic [29-32], and burnout and depressive symptoms are particularly prevalent [30,33,34]. Sleep problems due to shift work are associated with higher likelihoods of PTSD and depressive symptoms [27,35], whereas fatigue caused by sleep deprivation is linked to elevated rates of injury and medical errors for EMS providers [36]. In addition to this, exposure to MI and PMIEs further exacerbates these underlying vulnerabilities [37]. Health care workers facing PMIEs experience more severe depressive symptoms, burnout, sleep problems, and anxiety [38,39], along with greater turnover rates [40].

Such exposure to PMIEs can be particularly exacerbated during health care service disruptions. During the COVID-19 pandemic, health care workers, including EMS providers, were exposed to severe PMIEs due to delays in patient acceptance caused by critical shortages of hospital beds and medical resources [20,37], resulting in a significant increase in the prevalence of burnout and depressive symptoms [32,41,42]. In this study, EMS providers exposed to HPDE reported significantly higher rates of burnout, depressive symptoms, and sleep problems relative to those without such exposure.

Although the COVID-19 pandemic and the 2024 government–medical community dispute arose from different causes, both situations are comparable in that they led to the identical consequence of medical institutions being unable to accept patients due to health care service disruptions. This suggests that PMIEs, which inflict critical impacts on EMS providers, are not unique phenomena occurring only during infectious disease outbreaks. Regardless of the underlying cause, if a health care service disruption occurs, EMS providers can be exposed to severe PMIEs at any time. Therefore, to minimize psychological damage to EMS providers during unavoidable health care service disruptions, it is required to establish a systematic framework capable of early detection and response to PMIEs. While EMS providers inherently work in unpredictable and uncontrollable environments, exposure to HPDEs and PMIEs driven by these structural factors can cause irreversible damage to their mental health. Therefore, to protect the mental health of EMS personnel and prevent turnover, proactive and systematic organizational psychological intervention strategies—rather than reactive measures—are essential. Specifically, an organizational safety net must be established to proactively incorporate moral injury screening tools—such as the Moral Injury Events Scale and Moral Injury and Distress Scale for tracking event exposure [43,44], alongside the Moral Injury Outcome Scale and Moral Injury Symptom Scale-Healthcare Professionals for clinical symptom assessment [45,46]—into the routine health examinations of EMS providers to identify high-risk individuals early [47] and immediately provide specialized psychological counseling effective in addressing guilt and shame to those identified. Such organizational interventions will contribute to reducing the prevalence of mental disorders and enhancing the professional sustainability of EMS providers.

Although this study holds significant academic and clinical value as an investigation into the mental health outcomes of EMS providers exposed to HPDEs during government–medical community disputes, and provides policy-relevant evidence to improve emergency medical system delivery and the working conditions of EMS providers in scenarios involving health care service disruptions, several limitations must be acknowledged. First, despite extensive efforts to increase participation, including collaboration with firefighter unions, the response rate remained low at approximately 6% of all EMS providers. This voluntary participation–based sampling method can introduce selection bias; it is possible that providers experiencing higher levels of occupational stress or psychological distress participated more actively in the survey, potentially leading to an overestimation of the prevalence of mental health problems. Additionally, the geographic concentration in metropolitan areas (Seoul and Gyeonggi Province) limits the generalizability of the results to the entire domestic EMS provider population. Second, reliance on self-reported data introduces potential recall bias. In particular, measuring HPDE exposure with a single self-reported item restricts reliability and validity, and the possibility of misclassification due to respondents’ subjective memory and personal interpretation cannot be ruled out. Although this study used an intuitive item to rapidly capture urgent health care service disruption scenarios, it is limited by its inability to reflect more precise, multidimensional measurements such as the frequency, duration, and intensity of HPDE exposure. Third, the retrospective cross-sectional design prevents definitive conclusions about causal relationships between HPDE exposure and subsequent mental health outcomes. In future studies, the introduction of objective and multidimensional measurement tools to supplement the single item is needed, alongside large-scale, nationally representative studies to generate robust evidence for emergency care and working environment policy improvements.

In conclusion, regardless of the cause, unavoidable health care service disruptions can expose EMS providers to severe psychological crises, including MI. To prevent and minimize such occupational damage, it is urgently required to officially include moral injury screening tests, such as the Moral Injury Outcome Scale [45,47], in the routine health examinations of EMS providers and establish customized psychological treatment and management programs that support healing and recovery from MI.

## Supplementary material

10.2196/90130 Multimedia Appendix 1 Full multivariable logistic regression models for mental health outcomes, including health care–related patient deterioration experience and covariates, among emergency medical service providers in South Korea, November-December 2024.

10.2196/90130 Checklist 1 STROBE checklist.

## Abbreviations

CRB: citizen-related burnout
EMS: emergency medical service
HPDE: health care–related patient deterioration experience
MI: moral injury
PB: personal burnout
PMIE: potentially morally injurious event
PTSD: posttraumatic stress disorder
VIF: variance inflation factor
WRB: work-related burnout
