# Supplementary material for: Associations of Health Care System Deficiency–Related Patient Deterioration With Burnout, Depressive Symptoms, and Sleep Problems Among Emergency Medical Service Providers: Cross-Sectional Study
Source: JMIR Public Health Surveill. 2026 Jul 27;12:e90130. doi: 10.2196/90130 (PMC13404935; doi:10.2196/90130)
Supplement: Multimedia Appendix 1 [file publichealth-v12-e90130-s001.docx]

Multimedia Appendix 1. Full multivariable logistic regression models for mental health outcomes, including healthcare-related patient deterioration experience (HPDE) and covariates, among emergency medical service providers in South Korea, November–December 2024.

| **Potential Risk Factors** | | **Personal Burnout,**  **AOR (95% CI)** | **Work-related Burnout,**  **AOR (95% CI)** | **Citizen-related Burnout,**  **AOR (95% CI)** | **Depressive Symptoms,**  **AOR (95% CI)** | **Sleep Problems, AOR (95% CI)** |  |
| --- | --- | --- | --- | --- | --- | --- | --- |
|  |  |  |  |  |  |  |  |
| HPDE | |  |  |  |  |  |  |
|  | No | Ref | Ref | Ref | Ref | Ref |  |
|  | Yes | 2.05 (1.46, 2.87) | 2.66 (1.86, 3.78) | 2.74 (1.95, 3.86) | 2.22 (1.36, 3.60) | 1.63 (1.17, 2.27) |  |
| Sex | |  |  |  |  |  |  |
|  | Male | Ref | Ref | Ref | Ref | Ref |  |
|  | Female | 1.72 (1.20, 2.46) | 1.65 (1.15, 2.38) | 1.23 (0.86, 1.77) | 1.62 (1.06, 2.47) | 1.31 (0.92, 1.85) |  |
| Age | |  |  |  |  |  |  |
|  | 20-29 | Ref | Ref | Ref | Ref | Ref |  |
|  | 30-34 | 0.91 (0.55, 1.48) | 1.18 (0.71, 1.96) | 1.05 (0.63, 1.73) | 2.04 (1.04, 4.02) | 1.17 (0.72, 1.90) |  |
|  | 34-39 | 0.68 (0.37, 1.24) | 0.81 (0.44, 1.50) | 0.54 (0.29, 1.00) | 1.86 (0.83, 4.15) | 0.77 (0.42, 1.39) |  |
|  | 40-44 | 0.79 (0.42, 1.50) | 1.01 (0.52, 1.92) | 0.55 (0.29, 1.06) | 2.19 (0.93, 5.10) | 0.90 (0.48, 1.69) |  |
|  | ≥45 | 0.75 (0.34, 1.64) | 0.92 (0.41, 2.06) | 0.72 (0.33, 1.58) | 1.57 (0.55, 4.44) | 0.69 (0.32, 1.52) |  |
| Marital status | |  |  |  |  |  |  |
|  | Single | Ref | Ref | Ref | Ref | Ref |  |
|  | Married | 1.28 (0.85, 1.94) | 1.14 (0.75, 1.74) | 1.60 (1.05, 2.44) | 0.82 (0.49, 1.37) | 0.87 (0.58, 1.31) |  |
|  | Divorced/Separated/Widowed | 2.99 (0.87, 10.31) | 1.34 (0.40, 4.42) | 3.59 (1.04, 12.34) | 10.09 (2.78, 36.56) | 1.95 (0.60, 6.35) |  |
|  | Declined to answer | 0.98 (0.21, 4.57) | 3.44 (0.71, 16.56) | 3.55 (0.75, 16.75) | 0.59 (0.06, 5.33) | 2.63 (0.55, 12.44) |  |
| Household size | |  |  |  |  |  |  |
|  | 1 person | Ref | Ref | Ref | Ref | Ref |  |
|  | 2 persons | 1.05 (0.65, 1.69) | 1.16 (0.71, 1.89) | 0.82 (0.51, 1.34) | 0.80 (0.43, 1.48) | 1.33 (0.83, 2.14) |  |
|  | 3 persons | 1.04 (0.65, 1.67) | 1.05 (0.65, 1.69) | 0.74 (0.46, 1.19) | 1.21 (0.68, 2.13) | 0.88 (0.55, 1.40) |  |
|  | 4 persons | 0.99 (0.60, 1.65) | 0.77 (0.46, 1.30) | 0.76 (0.46, 1.28) | 1.26 (0.67, 2.36) | 1.19 (0.72, 1.96) |  |
|  | ≥5 persons | 0.63 (0.28, 1.43) | 1.07 (0.47, 2.4) | 0.92 (0.41, 2.05) | 0.97 (0.34, 2.75) | 1.09 (0.49, 2.42) |  |
| Education level | |  |  |  |  |  |  |
|  | High school or less | Ref | Ref | Ref | Ref | Ref |  |
|  | College | 0.73 (0.30, 1.76) | 1.56 (0.60, 4.03) | 2.37 (0.93, 6.01) | 0.46 (0.16, 1.28) | 2.07 (0.80, 5.35) |  |
|  | Graduate school | 3.42 (0.71, 16.40) | 5.20 (1.14, 23.67) | 3.81 (0.95, 15.24) | 1.41 (0.33, 5.93) | 5.99 (1.42, 25.19) |  |
|  | Declined to answer | 0.34 (0.02, 5.94) | 1.5 (0.07, 28.44) | 0.62 (0.03, 10.98) | 0.52 (0.02, 11.13) | 1.44 (0.09, 22.60) |  |
| Household income | |  |  |  |  |  |  |
|  | <40 million KRW | Ref | Ref | Ref | Ref | Ref |  |
|  | 40–49.99 million KRW | 0.81 (0.46, 1.42) | 0.98 (0.55, 1.74) | 0.80 (0.45, 1.41) | 0.57 (0.28, 1.14) | 1.29 (0.74, 2.24) |  |
|  | 50–59.99 million KRW | 0.67 (0.38, 1.17) | 0.87 (0.49, 1.56) | 0.98 (0.54, 1.74) | 0.85 (0.43, 1.69) | 1.54 (0.88, 2.70) |  |
|  | 60–69.99 million KRW | 0.66 (0.35, 1.27) | 0.76 (0.39, 1.47) | 0.69 (0.35, 1.33) | 0.69 (0.31, 1.54) | 1.42 (0.75, 2.71) |  |
|  | ≥70 million KRW | 0.68 (0.39, 1.21) | 0.86 (0.48, 1.55) | 0.78 (0.43, 1.39) | 0.64 (0.32, 1.30) | 1.12 (0.63, 1.97) |  |
|  | Declined to answer | 0.41 (0.19, 0.85) | 0.37 (0.16, 0.81) | 0.38 (0.17, 0.82) | 0.57 (0.22, 1.47) | 0.78 (0.37, 1.64) |  |
| Job position | |  |  |  |  |  |  |
|  | Ambulance crew | Ref | Ref | Ref | Ref | Ref |  |
|  | Ambulance driver | 0.80 (0.54, 1.19) | 0.59 (0.39, 0.89) | 0.83 (0.56, 1.23) | 0.80 (0.04, 1.34) | 0.93 (0.74, 1.44) |  |
|  | Other^a^ | 0.32 (0.06, 1.56) | 0.31 (0.06, 1.55) | 0.23 (0.04, 1.15) | 0.08 (0.00, 1.52) | 0.32 (0.23, 6.74) |  |
| Current assignment | |  |  |  |  |  |  |
|  | General EMS unit | Ref | Ref | Ref | Ref | Ref |  |
|  | Special EMS unit | 1.35 (0.94, 1.88) | 1.04 (0.74, 1.46) | 0.83 (0.59, 1.16) | 1.08 (0.71, 1.63) | 1.03 (0.74, 1.44) |  |
|  | EMS command and control center | 2.28 (0.42, 12.43) | 2.41 (0.43, 13.39) | 0.82 (0.15, 4.37) | 4.87 (0.53, 44.24) | 1.26 (0.23, 6.74) |  |
| Work type | |  |  |  |  |  |  |
|  | Day shift | Ref | Ref | Ref | Ref | Ref |  |
|  | Shift work (3 Teams, 2 Shifts) | 0.47 (0.08, 2.69) | 0.52 (0.08, 3.11) | 0.16 (0.02, 0.95) | 0.10 (0.01, 1.67) | 0.50 (0.08, 2.93) |  |
|  | Other shift work^b^ | 0.49 (0.08, 2.79) | 0.65 (0.11, 3.87) | 0.18 (0.03, 1.07) | 0.11 (0.01, 1.80) | 0.44 (0.07, 2.59) |  |
| Work region | |  |  |  |  |  |  |
|  | Rural | Ref | Ref | Ref | Ref | Ref |  |
|  | Urban | 2.41 (1.55, 3.72) | 3.37 (2.15, 5.31) | 2.10 (1.35, 3.26) | 1.96 (1.18, 3.26) | 2.18 (1.42, 3.35) |  |
|  | Sub-urban | 1.73 (1.26, 2.39) | 1.85 (1.33, 2.56) | 1.74 (1.26, 2.40) | 1.25 (0.82, 1.89) | 1.58 (1.15, 2.17) |  |
| HL chi-square | | 3.76 | 2.28 | 6.82 | 17.63 | 2.98 |  |
| P value | | 0.88 | 0.97 | 0.56 | 0.02 | 0.94 |  |
| c-statistic | | 0.73 | 0.76 | 0.73 | 0.74 | 0.69 |  |
| AOR, Adjusted odds ratio; CI, Confidence interval; HL, Hosmer-Lemeshow; HPDE, Healthcare system-oriented patient deterioration experience; EMS, Emergency medical services. | | | | | | |  |
| ^a^Other includes tasks such as report reception, counseling, and administrative duties. | | | | | | |  |
| ^b^Other shift work includes shift work (4 team, 2 shifts) | | | | | | |  |
